# High particulate matter emission from additive-free Natural American Spirit cigarettes

**DOI:** 10.1186/s40064-016-3635-x

**Published:** 2016-11-11

**Authors:** Yvonne Iffland, Ruth Müller, David Groneberg, Alexander Gerber

**Affiliations:** Center for Health Sciences, Institute of Occupational, Public and Environmental Medicine, Goethe University Frankfurt am Main, Frankfurt am Main, Germany

**Keywords:** Particulate matter, Indoor air pollution, Cigarettes

## Abstract

**Background:**

Involuntary exposure to health-threatening environmental tobacco smoke (Combined Mainstream and Side-stream Smoke, CMSS) is a worldwide problem, causing premature death of thousands of people. CMSS consists of particulate matter (PM), one of the main sources of indoor air pollution. PM constitutes a considerable health risk for passive smokers. It is important to inform the public about brand-specific differences in CMSS-associated PM, especially in the case of brands without additives, which are therefore promoted as natural and less health-threatening.

**Methods:**

Mean concentrations and the area under the curve of PM_10_, PM_2.5_ and PM_1_ generated by Natural American Spirit cigarettes without additives and the 3R4F standard research cigarette (University of Kentucky, USA) were measured, analyzed and compared with each other. An automatic environmental tobacco smoke emitter was used to smoke 100 cigarettes, 20 of each brand, according to a standardized smoking protocol.

**Results:**

This study could show that CMSS-associated PM released from tobacco brands without additives, which are therefore promoted as natural and less harmful, are higher than expected

**Conclusions:**

It is highly improbable that Natural American Spirit tobacco products are a less harmful choice—at least not for passive smokers as this study could show. We conclude, the CMSS-associated PM level of every single customized brand should be measured because the origin of the tobacco and not the amount of CO, tar and nicotine (given as product information) seem to be responsible for the brand-specific PM release. This data is urgently needed to adequately inform the public about CMSS-associated PM exposure and the related health risk especially for passive smokers.

## Background

Air pollution and its health hazards have been an important issue for researchers for decades. Besides other noxious components, airborne particulate matter (PM) contributes to ambient air pollution. PM consists of solid and liquid droplets floating through the air and can be inhaled by our lungs. The U.S. Environmental Protection Agency (EPA) distinguishes **PM**_**10**_ or coarse particles with a diameter of 2.5–10 µm and **PM**_**2.5**_ or fine particles with a diameter of 2.5 µm or less.^1^ PM_1_ defines ultrafine particles with a size of less than 1 µm.^2^ PM originates either from natural sources such as fire, volcano eruption or pollen or from human activities such as cooking, heating, industries, traffic or smoking (see footnote 1). After being inhaled PM causes severe consequences on human health in a dose–response manner, which has been demonstrated in several studies (Choudhury et al. 1997; Laden et al. 2006; Atkinson et al 2001; Bell et al. 2008; Brook et al 2010). Furthermore the toxicity of PM is size-depending. The smaller the diameter of the particles, the deeper they can reach into our respiratory system (Li et al. 2016). Particles with a size of less than 2.5 µm are even able to reach the alveoli, the gas exchange regions, where they might also be transferred to the bloodstream (Siponen et al. 2015). PM exposure is associated with e.g. cardiovascular, respiratory tract diseases, sudden infant death syndrome (SIDS), preterm birth and premature death (Bell et al. 2008; Jacquemin et al. 2015; Qiu et al. 2014; U.S. Department of Health and Human Services 1986; Lai et al. 2016).

PM levels can sometimes be higher in enclosed buildings than outdoors because PM concentrations are less diluted indoors (Umweltbundesamt 2014). People spend almost 87% of their daily lives inside (Klepeis et al. 2001). More than 50% of England´s households have at least one smoker. In some of these households smoking takes place in the presence of children (Albar et al. 2014). These findings also match for Germany (Brenner and Mielck 1993).

Combined Mainstream and Sidestream Smoke (CMSS) exposure causes death of 41,000 American adult non-smokers every year (King et al. 2014). Studies suggest that besides various other noxious substances in CMSS, PM itself is a health-threatening component of CMSS, leading to increased illness and deaths (Atkinson et al 2001; Bell et al. 2008; Brook et al. 2010; Sunyer and Basagaña 2001). Taking into account that the majority of people spend their time indoors, and many are involuntarily exposed to CMSS, we find it extremely relevant to investigate PM levels that occur by smoking inside. Moreover, we believe it is crucial to provide consumers with information not only about nicotine-, tar- and CO-content of their cigarette, but about the amount of PM that is released by it, as it presents a risk factor on its own.

The ToPIQ-II study tends to investigate brand-specific differences in the amount of PM emission of different tobacco products. Natural American Spirit cigarettes, produced by Santa Fe Natural Tobacco Company, are promoted to be additive-free and thus supposed to be natural and healthy tobacco products. The company targets “socially and environmentally conscious smokers” who want to stand out of the crowd (McDaniel and Malone 2007). Consumers believe in this promising advertisement and tobacco products, such as Natural American Spirit cigarettes, are gaining popularity (McDaniel and Malone 2007). 3 ^,^ 4 ^,^ 5 In this trial, PM emissions of Natural American Spirit cigarettes without additives were generated and then the CMSS exposure risk for passive smokers was analyzed.

## Methods

### Tobacco products

The four tobacco products, tested in present study, belong to the brand Natural American Spirit cigarettes, produced by Santa Fe Natural Tobacco Company. Natural American Spirit orange contained 0.4 mg nicotine, 3 mg tar and 4 mg CO per cigarette. The blue counterpart had a nicotine yield of 1 mg, a tar yield of 9 mg and a CO yield of 10 mg. The green type contained 0.8 mg nicotine, 7 mg tar and 8 mg CO and the yellow type, respectively, 0.6, 5 and 6 mg.^6^ These tobacco products were analyzed and compared with 3R4F standard research cigarettes,^7^ which have a nicotine content of 0.726 mg, a tar yield of 9.4 mg and a CO content of 11.9 mg. Reference cigarettes have been used in scientific studies for several years, e.g. in order to analyze their chemical or toxicological effects in comparison to commercial tobacco products.^8^

### Automatic environmental tobacco smoke emitter (AETSE)

This experiment is part of the ToPIQ-II study and therefore has the same setup as previously conducted studies (Wasel et al. 2015; Gerber et al. 2015a, b; Mueller et al. 2012). Developed and constructed by Schimpf-Ing, Trondheim Norway, the AETSE was placed in a 2.88 m^3^ glass chamber that serves as a defined indoor space. The AETSE was equipped with a 200 ml glass syringe, a microcontroller, a stepper motor, aluminum profiles, a dilution system and other mechanical devices. The originated PM levels were diluted in a ratio of 1:10 by the dilution system before being analyzed. Actual values are, respectively, ten times higher.

The glass syringe generated puffs of 40 ml volume through pushing and pulling. The stepper motor made it possible to evoke the automatic pushes and pulls of the syringe plunger. By pulling the syringe, inhalation was simulated. Correspondingly, by pushing the syringe, the inhaled CMSS was exhaled into the glass chamber through the hose system. Particularly, this procedure imitated the mainstream smoke. Between two puffs, the smoldering cigarette steadily produced side-stream smoke (SS) which together with mainstream smoke added up to CMSS that was measured constantly, every 6 s, by a laser aerosol spectrometer (Model 1.109, Grimm Co., Ainring, Germany). Placed in a separate room, the glass chamber was equipped with rubber gloves. Using these gloves smoking cycles were started and finished from the outside of the chamber without opening the chamber’s door so that the examiner was not exposed to CMSS at any time. Additionally, turbulences and ventilations in the chamber by opening the door could be minimized.

### Smoking protocol

**Fig. 1:**
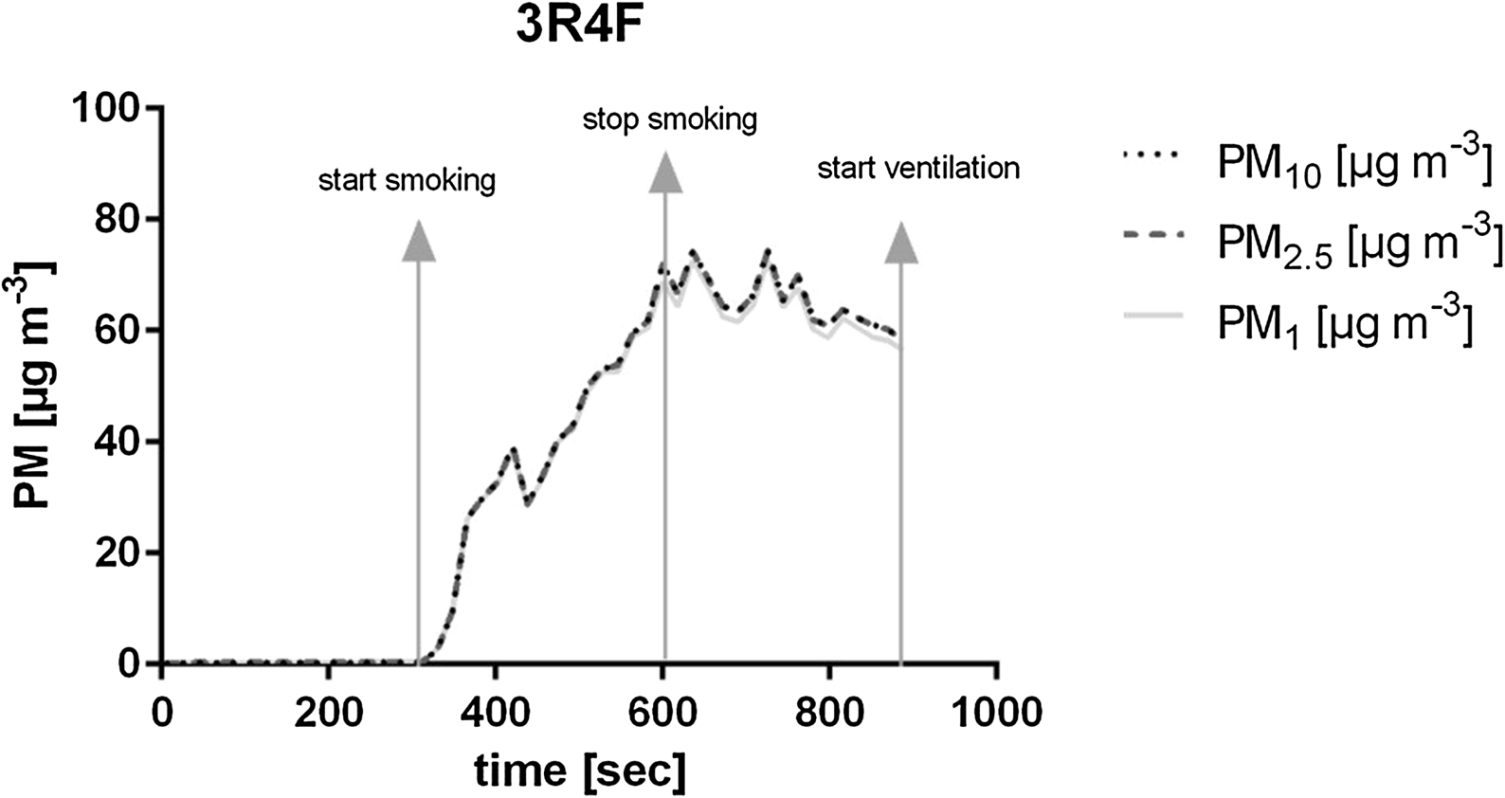
Sample measuring cycle

In order to smoke cigarettes in a standardized, reproducible and reliable way, the following four-phase smoking protocol was used: (1) the pre-igniting phase, (2) the combustion phase, (3) the post-combustion phase and (4) the suction phase (Fig. 1).

The *pre*-*igniting phase* lasted 5 min. During this time pre-PM levels in the glass chamber are measured in order to receive baseline PM levels. After the 5 min a cigarette is manually lit by the examiner from the outside of the glass chamber using the provided gloves. In the *combustion phase* the cigarette is smoked until 0.5 cm remain to the filter. Natural American Spirit cigarettes needed 13 puffs, each lasting 3 s. 3R4F needed 8 puffs with the same duration. This results in a combustion time of 7 min and 35 s for Natural American Spirit cigarettes and 4 min and 55 s for 3R4F. The volume of each puff is 40 ml and the time in-between two puffs is 24 s. The first puff needed to be a double puff to avoid the extinguishing of the cigarette. At the end of this phase the cigarette was manually extinguished in a bowl filled with water. Subsequently, the 5-min post-combustion phase began. Afterwards a 5-min suction phase followed in order to clean the air in the glass chamber for the next cycle.

### Data processing and analysis

Each smoking cycle was analyzed for the area under the curve (AUC) and the mean concentration (C_mean_) of PM_10_, PM_2.5_ and PM_1_, using the tenfold values detected by the laser aerosol spectrometer. To estimate the reliability of single measurements, the percentage AUC of all peaks greater than threefold of the CMSS measured during the post-combustion phase was calculated in five randomly selected cigarettes per brand. A percentage greater than 22% was set as an internal limit, indicating that the impact of mainstream smoke is too high. Among the 20 cigarettes per PM class and product, outliers were identified with the Iglewicz and Hoaglin's robust test for multiple outliers (one outlier for Natural American Spirit orange could be detected and then excluded from the results). Subsequently, Gaussian normality of the AUC and C_mean_ per PM class and tobacco product was tested, applying the D’Agostino-Pearson test and a significance level of 0.05. Additionally, log-transformed AUC and C_mean_ of PM_10_, PM_2.5_ and PM_1_ with homogeneous variances (Bartlett’s test, p = 0.18) were tested for significant differences between the five tobacco products, using one-way ANOVA and Tukey posthoc tests. This statistical method was also applied to examine the influence of the combustion time in reference to the PM emission. Using the Spearman correlation test, physiological factors such as CO, nicotine and tar content were examined in respect to their influence on PM emission. All statistics were computed with the software Graph Pad Prism version 6.

## Results

**Fig. 2:**
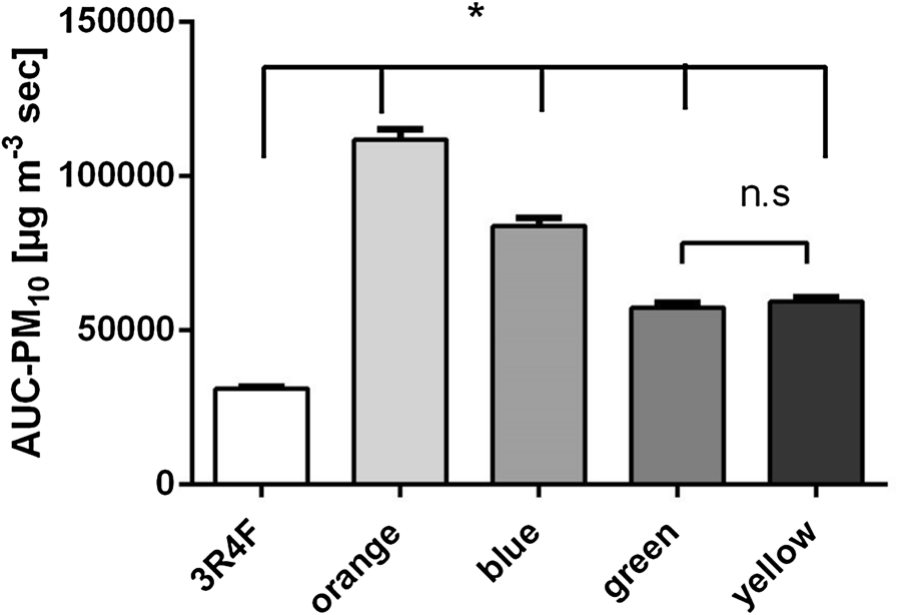
Comparison of the AUC of 3R4F and Natural American Spirit cigarettes (significance was assumed when p < 0.05; *significant, *ns* non-significant

**Fig. 3:**
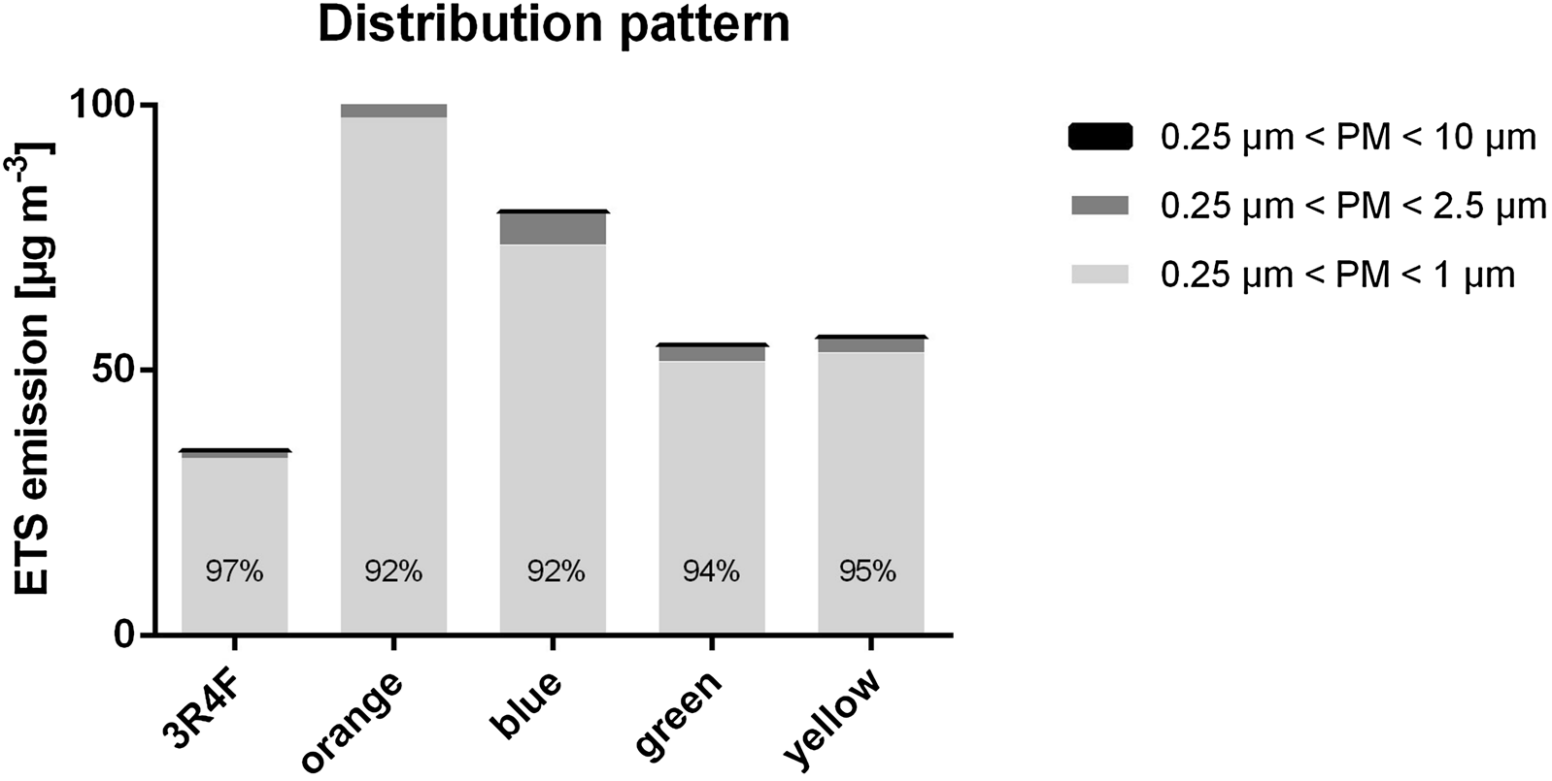
Scattering scheme of PM emissions in CMSS

The proportion of artificial peaks was highest for Natural American Spirit yellow with 14.6% (3R4F: 8.2%; orange: 4.6%; blue: 7.6%; green: 6.6%), but smaller than 22%. Thus it can be assumed that mainly SS was measured. A Gaussian distribution could be found for all PM values of each tested cigarette brand (p 3R4F = 0.46; p orange = 0.08; p blue = 0.69; p green = 0.27; yellow = 0.41). C_mean_ of PM_10_ and the AUC-PM_10_ of Natural American spirit orange are 1.3 times higher than C_mean_ of the blue type, 1.9 times higher than C_mean_ of the green type and 1.8 times higher than C_mean_ of the yellow type (Fig. 2; Table 1). The same relation between tobacco products applies for the C_mean_ of PM_2.5_ and PM_1_ and the AUC-PM_2.5_ and AUC-PM_1_. AUC levels of Natural American Spirit cigarettes are significantly higher than those of 3R4F, which is probably caused by the longer time of combustion. Whereas 3R4F cigarettes only needed 8 puffs, respectively, a combustion time of 4:55 min, all Natural American Spirit cigarettes burned 7:35 min (13 puffs). The relation between combustion time and CMSS emission was significant (p < 0.001). A specific scattering scheme for each cigarette could be detected (Table 2; Fig. 3). PM emission of Natural American Spirit orange mainly consists of PM_1_ (97.66 µg m^−3^ = 92.4%), only 7.6% (8.04 μg m^−3^) of PM_2.5_ and 0.02% (0.02 μg m^−3^) of PM_10_. PM concentrations of Natural American Spirit blue are divided into 92% (73.55 μg m^−3^) PM_1_, 7.4% (5.9 μg m^−3^) PM_2.5_ and 0.6% (0.14 μg m^−3^) PM_10_. PM emitted by Natural American Spirit green is also mainly composed by PM_1_ with 94.8% (53.18 μg m^−3^), and the remaining parts are distributed to PM_2.5_ with 5.1% (2.79 μg m^−3^) and PM_1_ with 0.1% (0.06 μg m^−3^). The distribution pattern is similar for Natural American Spirit yellow: PM_1_ 95.1% (53.18 μg m^−3^), PM_2.5_ 4.8% (2.7 μg m^−3^) and PM_10_ 0.01% (0.04 μg m^−3^).

**Table 1:** Overview of AUC and C_mean_ of all investigated cigarettes and their combustion time

Tobacco product	PM_10_		PM_2.5_		PM_1_		Time of combustion
	AUC PM_10_ (µg m^−3^ s) × 10	C_mean_ PM_10_ (µg m^−3^) × 10	AUC PM_2.5_ (µg m^−3^ s) × 10	C_mean_ PM_2.5_ (µg m^−3^) × 10	AUC PM_1_ (µg m^−3^ s) × _10_	C_mean_ PM_1_ (µg m^−3^) × 10	Sec
3R4F	30,952 ± 1420	34.59 ± 2.01	30,943 ± 2356	34.58 ± 2.01	30,051 ± 2513	33.61 ± 2.62	295
Orange	111,494 ± 15,503	105.9 ± 7.21	111,276 ± 15,390	105.7 ± 7.82	100,729 ± 11,201	93.17 ± 7.37	455
Blue	83,681 ± 12,257	79.59 ± 7.47	83,533 ± 11,696	79.45 ± 7.47	78,720 ± 6216	71.83 ± 11.67	455
Green	57,183 ± 5658	54.37 ± 4.19	57,115 ± 5658	54.31 ± 5.59	53,324 ± 4192	52.69 ± 5.47	455
Yellow	59,178 ± 7615	55.92 ± 6.26	59,133 ± 7615	55.88 ± 1.28	56,141 ± 5914	54.17 ± 2.17	455

**Table 2:** Distribution pattern of PM emissions

C_mean_ (_µg m_^−3^)	3R4F	Orange	Blue	Green	Yellow
0.25 µm < PM < 10 µm	34.59 ± 0.65	105.9 ± 3.39	79.59 ± 2.50	54.37 ± 1.59	55.92 ± 1.28
0.25 µm < PM < 2.5 µm	34.58 ± 0.65	105.7 ± 3.37	79.45 ± 2.50	54.31 ± 1.58	55.88 ± 1.28
0.25 µm < PM < 1 µm	33.47 ± 0.62	97.66 ± 2.66	73.55 ± 2.08	51.52 ± 1.41	53.18 ± 1.09
*Actual PM concentrations due to EPA´s definition*					
PM_1-EPA_ (<1 µm)	33.47 ± 0.62	97.66 ± 2.66	73.55 ± 2.08	51.52 ± 1.41	53.18 ± 1.09
PM_2.5-EPA_ (1–2.5 µm)	1.11 ± 0.03	8.04 ± 0.71	5.9 ± 0.42	2.79 ± 0.17	2.7 ± 0.19
PM_10-EPA_ (2.5–10 µm)	0.01 ± 0.065	0.02 ± 0.02	0.14	0.06 0.01	0.04

Taking the varying CMSS-associated PM amounts of different Natural American tobacco products into account, the question arises which factors do have an influence on the CMSS emissions, remembering that Natural American Spirit cigarettes are supposed to be free of additives (see footnote 6). Therefore correlations between nicotine-, tar- and CO-content and filling density were performed. Neither a correlation between nicotine-, tar- and CO-content and PM values nor filling density or tobacco weight and PM emission could be found (r = −0.04 for all correlations).

## Discussion

We were successful to demonstrate significant brand-specific differences regarding PM concentrations in CMSS. CMSS emissions are not predictable by CO-, tar- and nicotine-content, given as product information. They appear to depend on the origin of the tobacco and the brand-specific design of the tobacco product and its typical tobacco blend. This result implies that standardized brand-specific measures of CMSS emissions are inevitable for exposure risk assessment, especially when compared to a standard reference cigarette, and should be stated as product information on tobacco products.

### Brand-specific PM differences

Additive-free cigarettes, typically promoted as natural tobacco products, do not decrease the CMSS exposure risk for passive smokers. We state that CMSS emission is high and CMSS consists mainly of particles with a size of 0.1–1 µm (>90%). A possible explanation may be the impact of the tobacco, used in these cigarettes. If the tobacco differs, for example, in the amount of cellulose or contains different phytochemicals, this might have an effect on combustion. Wasel et al. (2015) examined PM emission released by L&M cigarettes with and without additives. Also in this trial C_mean_ of PM_2.5_ of the additive-free product were higher (576 µg m^−3^) when compared with the types containing additives (LM blue: 448 µg m^−3^; LM red: 547 µg m^−3^). Besides the higher PM emissions, additive-free Natural American Spirit tobacco products also reveal a potential health risk on passive smokers because of the distribution pattern of emitted fine particles. By far the highest part of PM in SS consists of PM_1_, which is most threatening on human health (Li et al. 2016). After extended research we believe that other than in the ToPIQ-II (Wasel et al. 2015; Gerber et al. 2015a, b; Mueller et al. 2012), differences between brands regarding PM emission in SS have not been investigated. Nevertheless, there are a few studies where CMSS-associated PM concentrations were measured. However, in those cases CMSS was not automatically generated by a machine but by human smokers (Martin et al. 1997; Nelson et al. 1997; Leaderer and Hammond 1991).

### Methodological limitations

Using the AETSE with a standardized smoking protocol enables us to generate reliable and reproducible PM levels. Nevertheless, it does not imitate the smoking behavior of a real smoker, even if it is possible to alter puff volume, volume flow rate, duration, breaks and repetitions. It is likely that different brands are smoked in different ways, for example due to nicotine, tar and CO content (Guerin 1996; Creighton 1976). If smokers draw longer, mainstream smoke increases. Respectively, if the time between two drags increases, CMSS increases as well. However, this method serves the aim to compare PM emission of different brands under the same standardized conditions without exposing humans to harmful CMSS. Before starting the experiments, cigarettes of each type included in the study were smoked until 0.5 cm remained to the filter. It appears that Natural American Spirit cigarettes needed more drags, in fact 13, to fulfill this criterion, whereas the reference cigarette could be smoked with only eight drags.

We have to admit, that our experiments where not performed with an internationally accepted and recognized smoking regime, such as the “ISO machine smoking regime” or the “Canadian Intense”. In fact, our regime is tailor-made and constitutes a mixture of both regimes. We followed the ISO Intense in puff frequency, but reduced the puff volume. This was due to several reasons: First, the technical limitations of our AETSE, which had to be constructed with low financial opportunities, did not allow us to use one of the recognized smoking regimes. Furthermore, most internationally accepted protocols have been heavily criticized (Hammond et al. 2007). Other research groups have reconsidered and modified parameters as well (Baker 2002).

It should be mentioned though, that we were not primarily interested in the absolute amounts of C_mean_ and AUC PM_2.5_ of additive free Natural American Spirit cigarettes. All absolute data remain imprecise, as individual smoking behaviors- and conditions vary in countless ways. No smoking protocol is arguably able to exactly imitate human smoking behavior with all its inter- and intra-individual variations in a realistic way. However, the aim of our ToPIQ-II study is to compare different brands and tobacco products with the 3R4F standard research cigarette in terms of their PM emission, when smoked in a standardized way (Gerber et al. 2015a, b). We believe, our protocol is reasonable for this purpose. Nonetheless, we cannot compare our results with finding of other groups, which is admittedly a methodological weakness.

## Conclusions

Besides various additives and the well known toxic and carcinogenic compounds in tobacco products, CMSS-associated PM constitutes an independent health hazard. The smaller the particles are, passive smokers are exposed to, the more deeply they can be inhaled and even penetrate into the blood stream. Depending on their surface and solubility, particles absorb gasses and vapors (e.g. carzinogenic benzopyrenes), permitting their transport in distal lung areas and even to the gas exchanging regions of the Lung. Thus, even if we are not able to conduct a toxicological and chemical analysis of SS with our technical possibilities, it can be assumed that significant differences in PM emissions matter. The release of PM by Natural American Spirit cigarettes was shown to be significantly (2–3 times) higher than that of 3R4F reference cigarettes, when smoked in the same standardize way, using our protocol.

Even if Santa Fe foregoes using additives during the production process of their cigarettes, their tobacco products still evoke high PM emission in SS, which reveals a health hazard to passive smokers. We want to illuminate the impact of these differences on the burden of PM-exposure on passive smokers.

## Abbreviations

CMSS: Combined Mainstream and Sidestream Smoke
SS: side-stream smoke
AETSE: automatic environmental tobacco smoke emitter
PM: particulate matter
C_mean_: mean concentration
AUC: area under the curve
CO: carbon monoxide
SIDS: sudden infant death syndrome

## Footnotes

1 EPA US, OAR, OAQPS. Particulate Matter | Air & Radiation | US EPA. 10.09.2015. http://www3.epa.gov/pm/. Accessed 19 Jan 2016.

2 Green Facts. Air Pollution Particulate Matter. http://www.greenfacts.org/en/particulate-matter-pm/level-2/01-presentation.htm. Accessed 16 Feb 2016.

3 Fay JE. "All Natural" concept evaluation. https://industrydocuments.library.ucsf.edu/tobacco/docs/#id=yrcx0084. Accessed 21 Jan 2016.

4 Gurry N. All natural concept product test. https://industrydocuments.library.ucsf.edu/tobacco/docs/#id=nkkl0106. Accessed 21 Jan 2016.

5 Farrell B. The "Green" Cigarette? http://www.plentymag.com/features/2008/12/the_green_cigarette_print.php. Accessed 21 Jan 2016.

6 Santa Fe Natural Tobacco Company. Natural American Spirit® | SFNTC. https://www.naturalamericanspirit.de/. Accessed 20 Jan 2016.

7 University of Kentucky. CTRP - Center for Tobacco Reference Products. 16.01.2016. https://refcig.uky.edu/client/index.html. Accessed 20 Jan 2016.

8 Reference products used in tobaccoand smoke analyses. Tobacco Journal International 2013.
